# Obituary

**Published:** 1895-06

**Authors:** 


### Obituary.

DR. JOHN W. BYRON, chief of the bacteriological department of the Loomis laboratory, died recently in New York, aged 35 years, from tuberculosis, contracted, it is believed, while working in his laboratory.

A year ago, when he sailed for Europe, he told his friends, speaking in the coolest manner possible, that he had by accident received some of the bacilli of tuberculosis while experimenting with the culture of the germs, and that they were playing havoc with his lungs. He added that they might kill him, but that he had an idea that a remedy was possible and that he was going to Europe to get it.

Sadly enough it proved otherwise, and he returned last Fall knowing that he was doomed. He continued his work, however, as chief of the bacteriological department of the Loomis laboratory until a very few days before his death. Dr. Byron was one of the most eminent specialists in this study in this country, and was fearless in exposing himself to diseases which are the result of infectious bacilli. He gained a national reputation in 1892 by going to Swinburne Island when the cholera patients were quarantined there, and staying until the last victim had recovered.

The work of the bacteriologist among the virulent germs of loathsome diseases, it is shown by Dr. Byron's death, is attended by grave risks, and his heroic self-sacrifice cannot fail to lift him and his professional comrades up to a higher plane in the estimation of the world at large, for their sacrifice is on behalf of suffering humanity.

